# Radiographic Quality of Root Canal Obturation Performed By Fifth Year Students of Hamadan Dental School 

**Published:** 2017

**Authors:** Amir Eskandarloo, Hamed Karkehabadi, Seyedeh Zeinab Hoseini Hashemi, Masoumeh Ahmadi, Seyedeh Sareh Hendi

**Affiliations:** a *Department of Radiology, Dental School, Hamedan University of Medical Science, Hamadan, Iran; *; b *General Dentist, Hamadan, Iran; *; c * Research Committee, Department Of Endodontics, Dental School, Isfahan University Of Medical Science, Isfahan, Iran*

**Keywords:** Dental Students, Quality Control, Root Canal Obturation, Root Canal Therapy

## Abstract

**Introduction:**

The aim of this study was to assess the radiographic technical quality of root canal therapy performed by fifth year students of Dental School of Hamadan University of Medical Sciences from 2015 to 2016.

**Methods and Materials:**

Four hundred and seventy records of root canal therapies were evaluated. Records with graphies taken as initial, master apical file (MAF), master apical cone (MAC) and final radiographs were included in the study and records of patient younger than 16 years and older than 68 years were excluded from further investigations. Lastly, 432 teeth were selected. Obturation length, canal tapering, quality and density of filling material were the variables investigated in the present study. Two independent investigators examined the radiographies using a magnifying lens (×2) and x-ray viewer. Data were analyzed using chi-square test.

**Results:**

The technical quality of root filling performed by undergraduate dental students was classified as acceptable in 10.4% of cases. Moreover, 70.8% of teeth had adequate filling, 17.1% were underfilled and 12% were overfilled. The three groups were significantly different in terms of working length and taper quality. One hundred ninety four (44.9%) records had adequate taper and 109 (25%) records had adequate density. There was a significant association between teeth location and the length of obturation so that the probability of a successful treatment was higher in maxillary teeth. Furthermore, the rate of a proper length of obturation was higher among incisors than that of premolars and molars.

**Conclusion:**

The technical quality of root canal therapy performed by dental students in Hamadan University of medical sciences is not as acceptable as it should be. One of the most important factors in this regard is a high student/professor ratio.

## Introduction

Root canal therapy is regarded as one the most important tasks that a dentist should perform during his/her career [1]. In recent years, endodontic practice has considerably progressed in all aspects [2]. Although, in more than 94 percent of cases, dentists are able to carry out root canal therapy successfully [3], there are several determinants factors in outcome assessment that should be taken into account with the technical quality of obturation being the most important one [4]. Radiographs obtained before and after the implementation of the root canal therapy are normally used for assessing the success of treatment [5, 6]. The technical quality of root obturation is a function of several factors, including the distance between the filling material and the radiographic apex, the density of filling material and canal taper [7]. It has been reported by several studies that the distance between the filling materials and radiographic apex should not be higher than 2 mm [8]. Moreover, the extrusion of root filling materials out of the apex can threat the treatment success. Furthermore, a proper obscuration free of any void will reduce the risk of following periapical diseases [9]. Similarly, it has been stressed by many studies that when a filled root has a void in middle third or apical regions, tooth survival would significantly decrease [9]. The importance of root canal tapering has been fully discussed by Young *et al.* [10] and Buchanan [11]. It has been demonstrated by many studies that the size of taper has a significant impact on the removal of debris and smear layer from the root canal walls [10, 11]. 

Previously, many studies have attempted to investigate the technical quality of canal obturation performed by dentistry students, dentists and endodontists. Generally, they reported that the technical quality of obturation performed by dentistry students is not satisfactory [12-14]. Considering the importance of this issue in planning a better education for dental students, the present study was set to assess the effect of some factors on the technical quality of root obturation performed by dentistry students.

## Materials and Methods

This retrospective cross-sectional study was conducted in the Dentistry Faculty of Hamadan University of Medical Sciences during 2015-2016. The study was conducted on all cases who were referred to the dentistry clinic of the university. A total number of 470 cases were referred to the clinic during this period of time and were treated by 5^th^ year dental students and all cases had initial radiography for determining the working length and a final graph demonstrating the status of the tooth at the end of treatment were included in the study. Cases with age outside the range from 16 to 68 years old and those with radiographs with unsatisfactory quality were excluded from the study. Finally, based on these inclusion and exclusion criteria, 432 cases were selected for further analysis. Moreover, it was necessary for the cases to follow a standard treatment strategy approved by Endodontic Department of Dental School, Hamedan University of Medical Sciences. A standard method of treatment contained following steps; isolation with rubber dam, determination of canal length using bisector method, root canal preparation with K-files stainless steel (SybronEndo, USA), use of RC-Prep (Premier Dental Products, Philadelphia, USA) for lubricating the canal and passive step back method, washing the canal with normal saline or 2% chlorhexidine, use of calcium hydroxide in the cases that a multi-session treatment were needed and then filling the canal in accordance with lateral condensation technique using gutta-percha and ZOE-based sealer, and lastly, the temporary restoration of teeth with cavit.

In the next step, the number of teeth in the maxilla and mandible, the location of each tooth in the jaw, and number of roots and observable canals were determined. Each radiography was assessed by two independent university professors in a dark room using a high quality magnifier. All measurements were performed using a transparent ruler with the precision of 0.5 mm. In the cases that the results reported by two specialists differed from each other, an experienced radiologist was asked to reassess the controversial radiography. 

**Table1:** Types and location of the teeth investigated in the present study

**Tooth type**	**Location**		**Total (%)**
	**Maxilla (percent)**	**Mandible (percent)**	
**Incisor**	102 (35.4)	8 (5.6)	110 (25.5)
**Premolar**	130 (45.1)	71 (49.3)	201 (46.5)
**Molar**	56 (19.4)	65 (45.1)	121 (28)
**Total**	288 (100)	144 (100)	432 (100)

**Table 2:** Length of obturation according to the type teeth and their locations

**Obturation** ** length **	**Tooth type (%)**			**Total**	***P*** **-value**
	**Incisor**	**Premolar**	**Molar**		
**Short**	7 (6.4)	35 (17.4)	32 (26.4)	74 (17.1)	0.00
**Proper**	95 (86.4)	144 (71.6)	67 (55.4)	306 (70.8)	
**Long**	8 (7.3)	22 (10.9)	22 (18.2)	52 (12)	
**Total**	110 (100)	201 (100)	121 (100)	432 (100)	
**Obturation length**	**Tooth type (%)**			**Total**	***P*** **-value**
	**Maxilla**	**Mandible**			
**Short**	37 (12.8)	37 (25.7)		74 (17.1)	0.013
**Proper**	221 (76.7)	85 (59)		306 (70.8)	
**Long**	30 (10.4)	22 (15.3)		52 (12)	
**Total**	288 (100)	144 (100)		432 (100)	

The following criteria were used to categorize the radiographs; length of obturation; the distance between the upper surface of filling material and tooth apex. According to this definition, proper length of obturation was considered when the end of filling material was located at a distance lower than 2 mm from tooth apex. Moreover, long-term obturation was defined as location of filling material shorter than the radiographic apex and short-term obturation means the height of filling material was more than 2 mm shorter than the height of the radiographic apex. 

Assessment of obturation quality had two states; improper obturation containing void (the presence of void in filling materials was considered as a sign of improper obturation), proper obturation was defined as integrated obturation without any void. 

For root canal tapering a constant taper from coronal to apical aspect of the root and removal of debris and bacterial contamination were considered favorable [15]. 

It should be mentioned that in case of multi rooted teeth, we assessed every canal one by one, and if only one canal was of long or short obturation, the whole tooth was considered in long or short length group. Other variables were also assessed by the same approach. 

Finally, kappa coefficient was used for evaluating the inter-observer reliability and chi-square test was used for comparing variables to each other. All statistical tests were performed by SPSS software (version 18.0, SPSS, Chicago, IL, USA).

## Results

In the present study, a total number of 432 teeth including 110 incisors, 201 premolars, and 121 molars from both maxilla and mandible were investigated. Each radiograph was assessed by two independent specialists. In order to assess the inter-observer consistency, the KAPA coefficient was employed, which was equal to 0.89 which demonstrated an acceptable level of consistency between specialists. 

Table 1 shows detailed information about the teeth investigated in the present study. Table 2 represents data associated with the quality of obturation length. Obturation had a proper length in 70.8 percent of cases. Moreover, chi-square test revealed that there was a significant association between the tooth type and the quality of obturation so that the probability of a proper length of obturation was higher among incisors than that of premolars and molars. 

Moreover, Table 2 represents the obturation length according the location of teeth (in maxilla and mandible). The rightmost column of this table shows the result of chi-square test. 

**Table 3:** Taper quality according to the type teeth and their locations

**Taper quality**	**Tooth type (%)**			**Total**	***P*** **-value**
	**Incisor**	**Premolar**	**Molar**		
**Improper**	**Incisor**	**Premolar**	**Molar**	238 (55.5)	0.00
**Proper**	50 (45.5)	95 (47.3)	93 (76.9)	194 (44.9)	
**Total**	60 (54.5)	106 (52.7)	28 (23.1)	432 (100)	
**Taper quality**					
	
**Improper**	144 (50)	94 (65.3)		238 (55.1)	0.003
**Proper**	144 (50)	50 (34.7)		194 (44.9)	
**Total**	288 (100)	144 (100)		432 (100)	

**Table 4:** Quality of filling material density according to the type teeth and their locations

**Density quality**	**Tooth type (%)**			**Total**	***P*** **-value**
	**Incisor**	**Premolar**	**Molar**		
**Improper**	86 (78.2)	131 (65.2)	106 (87.6)	323 (74.8)	0.00
**Proper**	24 (21.8)	70 (34.8)	15 (12.4)	109 (25.2)	
**Total**	110 (100)	201 (100)	121 (100)	432 (100)	
**Taper quality**					
	
**Improper**	216 (75)	107 (74.3)		323 (74.8)	0.907
**Proper**	72 (25)	37 (25.7)		109 (25.2)	
**Total**	288 (100)	144 (100)		432 (100)	

Accordingly, there was a significant association between teeth location and the length of obturation so that the probability of a successful treatment was higher when teeth were located in the maxilla. 

As explained previously, in the present study, in addition to the obturation length, the quality of filling was also assessed. The associated results are presented in Table 3. According to the results, the association between the quality of filling and the type of teeth is of significant difference (*P*<0.05), so that the rate of proper and satisfactory filling was higher among incisors, followed by premolars and molars. According to the results presented in Table 3, the association between the tooth location and quality of filling is also significant (*P*<0.05). As evident in this table, the rate of successful filling was higher in maxillary teeth than mandibular ones. Table 4 also shows the results associated with the quality of filling material density. According to this table, in 74.8 percent of cases the quality of filling material density were not satisfactory. Moreover, this variable had the best results in premolars, in which 34.88% of cases had a successful treatment. The result of statistical test revealed that the association between tooth types and the quality of filling material density was significant (*P*<0.05). The results associated with the quality of filling material density and location of teeth are also presented in Table 4. Accordingly, the association between the quality of filling material density and the location of teeth was not significant (*P*>0.05). 

Finally, it should be noted that technical quality of only 45 teeth (10.4%) out of the 432 teeth investigated in the present study were found to be satisfactory. 

## Discussion

Root canal therapy is one the most important skills that general dentists should learn. Some general dentists are less motivated to take a part in treating molars because of their anatomical complexity and prefer to treat anatomically less complex teeth such as incisors [3]. Moreover, it has been stressed by many studies conducted in various regions of the world that the quality of treatments performed by general dentists are not as good as it should be [16-18]. Consequently, the present study was conducted in order to investigate the quality of root canal therapies performed by 5^th^ year dental student in 2014 and 2015. Further, in the present study, we utilized periapical radiographs instead of panoramic images to study the quality of treated teeth. 

In the present study, we considered three variable as indices for doing so; obturation length, quality of filling in terms of whether it contained voids or not, and canal tapering. These variables also have been utilized by several previous studies in order to accomplish the same aims [2, 4, 7, 19-21]. 

It was observed in the present study that most teeth (70.6 %) treated by the dental students had a proper obturation length, but it is difficult to compare these results with those reported by similar studies conducted in other universities all around the world, because of differences in the number of teeth and diversity of their types. However, the results are in line with the results reported by Ehsani *et al.* [22] and Er *et al.* [23]. In contrast, the successful treatment rate observed by the present study is higher than those of Elsayed *et al.* [2], Boltacz-Rzepkowska *et al.* [20], and Lupi-pegurler *et al.* [19] and lower than those of Moradi *et al.* [12] and Unal *et al.* [24]. 

It should be noted again that it is difficult to compare results reported by studies performed in various universities, however, in this section we attempted to provide some explanations for such differences. It was reported by Elsayed *et al.* [2] in Sudan that only 46% of students were able to provide patients with a proper obturation length, which is lower than what was observed in the present study. In Hamadan, students begin to work in clinic from the second semester of 4^th^ years, while Sudanese students begin to work in the clinic since the beginning of 6^th^ year. Moreover, the higher rate of proper obturation length obtained by Hamadani students compared with those reported by Boltacz-Rzepkowska *et al.* [20] and Lupi-pegurler *et al.* [19] can be due to different factors such as student’s skill, patient cooperation or attention of professors, and also checking the treatment process by providing several radiographs during root canal therapy in order to achieve the proper obturation. 

Periapical radiolucent lesion is another contributory factor that should be taken into account in this regard. Such a lesion can make it difficult for students to control the obturation length by damaging the strait of root canal. The different types of teeth also is another factor that may give rise to differences in results reported by various studies. 

One important factor which may significantly influence the success of root canal therapy is the professor/student ratio. The higher the ratio, the more the treatment success. As a proof for this statement, Moradi *et al.* [12] reported a ratio of 1:5 for the proportion of university professor to dental students, while in our faculty this ratio is 1:10, which consequently reduce the amount of time each professor spends for a student, and, as a result, the dental student would not train adequately. Similarly, the successful treatment rate reported from Unal *et al.* [24] is 92% which is much more than what we observed in the present study. It is interesting that the professor/student ratio reported by that study was 1:4. 

Furthermore, comparing various types of teeth, the obturation length was considered to be proper in 86.4%of incisors, 71.6% of premolars, and 55.4% of molars, emphasizing that the students are more capable in treating teeth with a simpler anatomical structure and more accessibility. Moreover, it was observed that the rate of teeth with a short obturation length was higher among molars (26.4%) than those in incisors and premolars. This can be due to the specific morphology of such teeth and also the difficulties in their access. 

Another important factor investigated by the present study was the quality of canal tapering. Accordingly, it was observed that 44.9% of cases had acceptable quality pertaining to this variable. The result was lower than those reported by Mokhtari *et al.* [25], Ehsani *et al.* [22], Balto *et al.* [7], Rafeek *et al.* [9] and Motamedi *et al.* [26], which can be due to the different methods used for cleaning and shaping of canals. For example, Balto *et al.* [7] reported that students used the step back method with K-files with 0.02 taper. Moreover, they described that students employed Gates-Glidden instruments, which can help them achieve a more desirable outcome. The same explanation can be offered for comparing the results of the present study and the study carried out by Motamedi *et al.* [26]. 

The quality of tapering was higher in anterior teeth. The results are in line with those of Barreishi Nusair *et al.* [4], Balto *et al.* [7], and Mokhtari *et al.* [25] and demonstrate that dentistry students from all around the world are struggling with treatment of teeth with a more complex anatomical structure. 

Moreover, 76.7% of teeth from maxilla and 59% of those from mandible had a proper length of obturation. The rate of proper root canal tapering was 50 and 34.7%, for maxillary and mandibular teeth, respectively. Fisher's exact test revealed that these differences are significant so that the rate of teeth with proper obturation length and root canal tapering were higher among teeth from maxilla than those from mandible. The results are in line with those of Elsayed *et al.* [2] and is mainly because of the difficulties students encounter in isolation of the mandibular teeth. 

The density of filling material is another important factor which is used in assessing the quality of root canal therapy. In radiographs it is regarded as proper when the density is uniform everywhere in the canal and compatible with the canal’s walls [19]. An improper density can increase the leakage and finally cause the treatment failure [27, 28]. It was observed in the present study that the density is acceptable in 25.2% of the cases. The rate was higher among incisors which is similar to the results of Mokhtari *et al.* [25], Moradi *et al.* [12], and Balto *et al.* [7] and mainly is because of their simple anatomical structures. These findings are in agreement with the results Rafeek *et al.* [9]. However, the rate is lower than those reported by studies such as Barrieshi *et al.* [20], Unal *et al.* [24], and Ehsani *et al.* [22]. As explained before, the professor/student ratio plays a detrimental role in this regard.

Overall, only 45 teeth (10.4% of all cases) were pleasant in terms of three factors of obturation length, density and tapering, which is similar to that of Rafeek *et al.* [9]. However, it is lower than those reported by other studies conducted by Elseyed *et al.* [2] and Balto *et al.* [7]. The method used by the students in root canal therapy, inadequate clinical supervision, and low precision in interpreting the radiographs after treatment could be some of the reasons why we observed such a low rate of proper root canal therapy in the present study. 

Lastly, in order for improving the quality of root canal therapies provided by dental students, it is recommended to verify the endodontic educational curriculum in a way that for example, the supervision of professors increase and the students spend more time in treating teeth with complex anatomical structures, particularly molars. 

## Conclusion

In conclusion, the technical quality of root canal therapy performed by dental students in Hamadan University of Medical Sciences is not as acceptable as it should be. One the most important factor in this regard is a high student/professor ratio.
